# *In vitro* studies reveal antiurolithic effect of *Terminalia arjuna* using quantitative morphological information from computerized microscopy

**DOI:** 10.1590/S1677-5538.IBJU.2014.0547

**Published:** 2015

**Authors:** A. Mittal, S. Tandon, S.K. Singla, C. Tandon

**Affiliations:** 1 Department of Biotechnology and Bioinformatics, Jaypee University of Information Technology, Waknaghat, Solan 173234, Himachal Pradesh, India; 2 Amity Institute of Molecular Medicine and Stem Cell Research (AIMMSCR), Amity University Uttar Pradesh, Noida; 3 Department of Biochemistry, Panjab University, Chandigarh – 160014, India; 4 Amity Institute of Biotechnology, Amity University Uttar Pradesh, Sector – 125, Noida, U.P., 201313 India

**Keywords:** Kidney, Calculi, Calcium Oxalate, Phytotherapy, Terminalia

## Abstract

**Purpose:**

For most cases, urolithiasis is a condition where excessive oxalate is present in the urine. Many reports have documented free radical generation followed by hyperoxaluria as a consequence of which calcium oxalate (CaOx) deposition occurs in the kidney tissue. The present study is aimed to exam the antilithiatic potency of the aqueous extract (AE) of *Terminalia arjuna* (*T. arjuna*).

**Materials and Methods:**

The antilithiatic activity of Terminalia arjuna was investigated in vitro nucleation, aggregation and growth of the CaOx crystals as well as the morphology of CaOx crystals using the inbuilt software ‘Image-Pro Plus 7.0’ of Olympus upright microscope (BX53). Antioxidant activity of AE of Terminalia arjuna bark was also determined in vitro.

**Results:**

Terminalia arjuna extract exhibited a concentration dependent inhibition of nucleation and aggregation of CaOx crystals. The AE of Terminalia arjuna bark also inhibited the growth of CaOx crystals. At the same time, the AE also modified the morphology of CaOx crystals from hexagonal to spherical shape with increasing concentrations of AE and reduced the dimensions such as area, perimeter, length and width of CaOx crystals in a dose dependent manner. Also, the Terminalia arjuna AE scavenged the DPPH (2, 2-diphenyl-1-picrylhydrazyl) radicals with an IC_50_ at 13.1µg/mL.

**Conclusions:**

The study suggests that Terminalia arjuna bark has the potential to scavenge DPPH radicals and inhibit CaOx crystallization in vitro. In the light of these studies, Terminalia arjuna can be regarded as a promising candidate from natural plant sources of antilithiatic and antioxidant activity with high value.

## INTRODUCTION

Urinary stones affect a large proportion of the population. Approximately 85% of urinary stones are calcium stones, which consist of oxalate and phosphate, either alone or in combination (1). The mechanisms involved in the formation of urinary stones are not fully understood but it is generally agreed that urinary lithiasis is a multifaceted process involving events leading to crystal nucleation, aggregation and growth of insoluble particles (2). Crystal growth and agglomeration may be due to supersaturation with respect to stone forming constituents or the presence of various inhibitory or stimulatory biomolecules or even pH (3). Urine is always supersaturated with common stone forming minerals, however, the crystallization inhibiting capacity of urine does not allow urolithiasis to happen in most of the individuals, whereas this natural inhibition is impaired in stone formers (4).

CaOx stones are mostly found in two different varieties, CaOx monohydrate (COM) or Whewellite, and CaOx dihydrate (COD) or Weddellite. COM, the thermodynamically most stable form, is observed more frequently in clinical stones than COD and has a greater affinity for renal tubular cells, thus responsible for the formation of stones in the kidney (5). CaOx monohydrate (COM) has been found to initiate mineralization followed by the deposition of CaOx dehydrate (COD) on it (6).

Development of modern techniques such as extracorporeal shock wave lithotripsy (ESWL), percutaneous nephrolithotomy (PCNL), or ureteroscopy (URS) have revolutionized surgical management of kidney stones in recent years but do not give satisfactory results as these techniques do not prevent the likelihood of new stone formation (7). Many medicinal plants have been used since ages to treat urinary stones though the rationale behind their use is not well established through systematic and pharmacological studies, except for some composite herbal drugs and plants (8).

In recent years, numerous studies describing the therapeutic properties of extracts from different parts of various medicinal plants have been developed. Indeed, the use of such extracts as complementary and alternative medicine has lately increased, and also serves as an interesting source of drug candidates for the pharmaceutical industrial research (9). *Terminalia arjuna*, belonging to the family Combretaceae, holds a reputed position in Ayurvedic system of medicine since ancient times (10). Experimental and clinical studies revealed the beneficial effects of this plant against all sorts of conditions of cardiac failure (11), dropsy, anti-infective (12), anti-asthmatic and for the treatment of rheumatoid arthritis and is traditionally used to prevent the formation of kidney stone.

The present study aims to exam the antilithiatic potency of the AE of *Terminalia arjuna* bark on crystallization of CaOx in vitro and antioxidant activity of the same.

## MATERIALS AND METHODS

### 

#### Plant

The dried bark of was purchased from Natural Remedies Pvt. Ltd., Bangalore, India. A collection of voucher specimen is available at the company.

#### Preparation of the AE of *Terminalia arjuna*

The dried fine powdered *Terminalia arjuna* bark was soaked in distilled water for 24 hours at 4°C. The extract was then filtered through muslin cloth followed by centrifugation at 10,000 rpm for 20 mins at 4°C and the filtrate was lyophilized to obtain the dried powder referred to as AE of *Terminalia arjuna* bark. The dried AE was stored in labeled sterile bottles and kept at −20°C. The various concentrations of the plant sample tested for their inhibitory potency were: 10µg/mL, 25µg/mL, 50µg/mL, 100µg/mL, 200µg/mL, 500µg/mL and 1000µg/mL which were prepared at the time of experiment.

#### DPPH radical scavenging Assay

The effect of AE of *Terminalia arjuna* bark on DPPH radical was estimated using the method of Liyana-Pathirana and Shahidi (13). A solution of 0.135 mM DPPH in methanol was prepared and 2.0mL of this solution was mixed with 2.0mL of extract in methanol. The reaction mixture was vortexed thoroughly and left in the dark at room temperature for 30 min. The absorbance of the mixture was measured spectrophotometrically at 517nm. Ascorbic acid was used as reference. The IC_50_ value was defined as the concentration (µg/mL) of extracts that scavenges the DPPH radicals by 50%. The ability to scavenge DPPH radical was calculated by the following equation: DPPH radical scavenging activity (%)=((Acontrol-Asample)/Acontrol) × 100 where Acontrol is the absorbance of DPPH radical plus methanol, Asample is the absorbance of DPPH radical plus sample extract/standard. Methanol was used as a blank.

#### CaOx crystallization assay

Stock solutions of 10.0mM calcium chloride (CaCl_2_) and 1.0mM sodium oxalate (Na_2_C_2_O_4_), containing 200mM sodium chloride (NaCl) and 10mM sodium acetate, were adjusted to pH 5.7. All chemicals were of the highest purity grade available. Before being used in crystallization experiments, solutions were filtered through Millex-GV membranes with a pore diameter of 0.22µm and warmed up to 37ºC in a water bath. For crystallization experiments, 1.5mL of the CaCl_2_ solution was transferred into a 10mm light path quartz cuvette and an additional 1.5mL of the Na_2_C_2_O_4_ solution was then added to final assay concentrations of 5.0mM for calcium and 0.5mM for oxalate, respectively. In the cuvette, the final solutions were stirred continuously and maintained at 37°C (14, 15).

Control experiment was performed with calcium/oxalate concentration ratio, i.e., 5.0/0.5mM/mM. After addition of the oxalate-containing solution, automated time-course measurements of optical density at 620nm (OD_620_) were performed, i.e., OD_620_ was recorded after every 60 seconds over 40 minutes. Experiments with added sample (100µL), where rates of nucleation and aggregation were considerably lower, had to be extended to 60 minutes. All crystallization experiments were performed at least in triplicate. Percentage inhibition produced by the AE was calculated as (1-(Tsi/Tsc)) X100, where Tsc was the turbidity slope of the control and Tsi the turbidity slope in the presence of the inhibitor.

#### CaOx crystal growth assay

Inhibitory activity against CaOx crystal growth was measured using the seeded solution-depletion as say described previously by Nakagawa and colleagues (16). Briefly, an aqueous solution of 10mM Tris-HCl containing 90mM NaCl was adjusted to pH 7.2 with 4N HC1. Stone slurry (1.5mg/mL) was prepared in 50mM sodium acetate buffer (pH 5.7). CaOx monohy drate crystal seed (from FTIR identified clinical kidney stones) was added to a solution containing 1mM CaCl_2_ and 1mM Na_2_C_2_O_4_. The reaction of CaCl_2_ and Na_2_C_2_O_4_ with crystal seed led to deposition of CaOx (CaC_2_O_4_) on the crystal surfaces, thereby decreasing free oxalate that is detectable by spectrophotometry at λ214nm. When AE is added into this solution, depletion of free oxalate ions will decrease if the test sample inhibits CaOx crystal growth. Rate of reduction of free oxalate was calculated using the baseline value and the value after 60 seconds incubation for 20 minutes, with or without test sample. The relative inhibitory activity was calculated as follows: % Relative inhibitory activity=((C-S)/C)x100, where C is the rate of reduction of free oxalate without any test sample and S is the rate of reduction of free oxalate with a test sample.

#### Image Analysis of Crystal Morphology

The stock solutions of 12.75mM CaCl_2_ and 2.25 mM Na_2_C_2_O_4_ were used to observe the size and morphology of the crystals and to verify the effect of incubation with the test material on CaOx crystal formation. 50µL of CaCl_2_ solution was added to wells in a 96-well plate. To each of the wells, 50µL of test sample and Na_2_C_2_O_4_ solution were added to obtain final concentrations of 4.25mM calcium and 0.75mM oxalate (17, 18). Each concentration of AE was prepared in triplicate. The plates were then incubated at 37ºC for 45 minutes. Crystal morphology was examined in five randomly selected fields at 100x magnification under an upright microscope (Olympus Corporation, Japan). Images were captured from different fields. The measurement parameters in terms of area, perimeter, length and width of CaOx crystals in the absence and presence of various concentrations of *Terminalia arjuna* AE were measured using the inbuilt software ‘Image-Pro Plus 7.0’ to show the efficacy of AE of *Terminalia arjuna*. Cystone was used as a positive control.

### Statistical analysis

Data were expressed as mean values of three independent experiments (each in triplicate) and analyzed by ANOVA (p<0.05) to estimate the differences between values of extracts tested using the software GraphPad Prism version 6.01.

## RESULTS

### 

#### DPPH radical scavenging Assay

**Figure 1:**
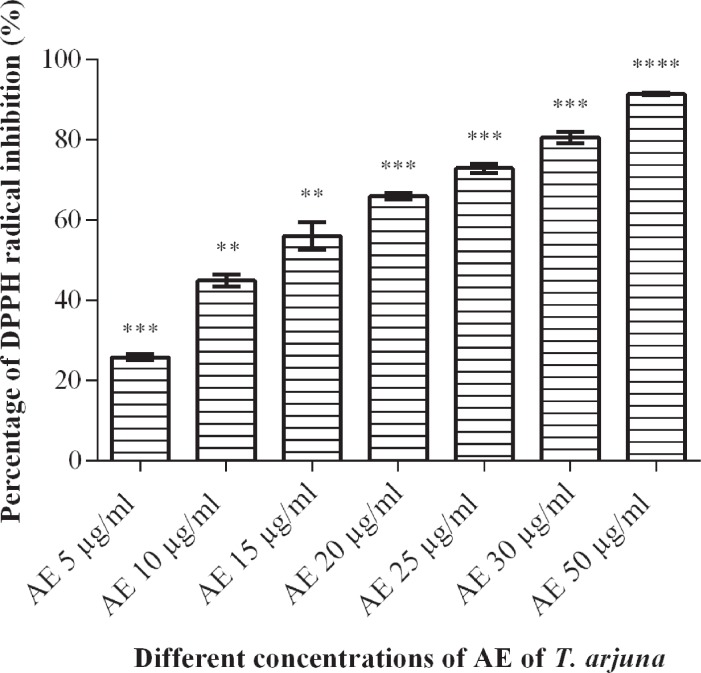
Effect of aqueous extract of Terminalia arjuna on DPPH radicals inhibition. Data are mean±S.D of three independent observations. **p<0.005, ***p<0.0005, ****p<0.0001

The antioxidant activity of AE of *Terminalia arjuna* was determined by measuring the DPPH radical scavenging activity. The AE of *Terminalia arjuna* bark displayed the DPPH radical scavenging activity. The ability to scavenge the DPPH radical increased with increasing concentrations of the extract in a dose-dependent manner as shown in Figure-1. The percentage of DPPH radical inhibition ranged from 25.82% at 5µg/mL to 93.87% at 50µg/mL. The AE caused scavenging of DPPH radical with IC_50_ value of 13.11µg/mL. The chemical, ascorbic acid was used as a standard and similarly inhibited DPPH with IC_50_ value of 5.84µg/mL.

#### CaOx crystallization assay

**Figure 2:**
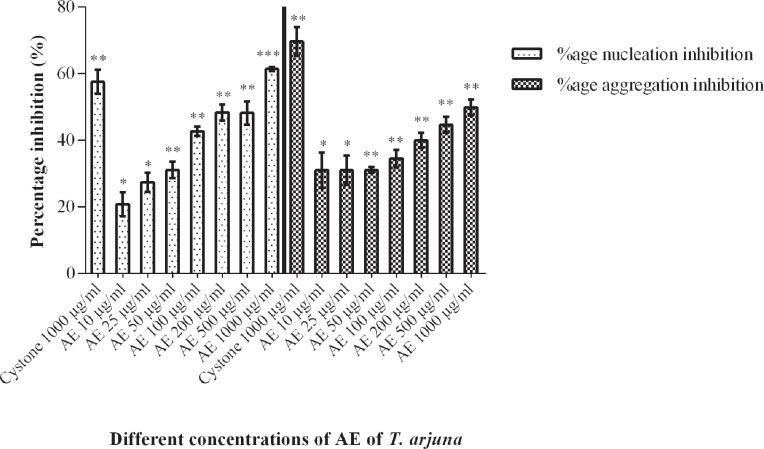
Effect of aqueous extract of Terminalia arjuna on nucleation and aggregation of calcium oxalate crystals. Data are mean±S.D of three independent observations. *p<0.05, **p<0.005, ***p<0.0005

At final concentrations of 5.0mmol/L calcium and 0.5mmoL/L oxalate, the CaOx crystallization inhibitory activity of *Terminalia arjuna* AE increased with increasing concentrations of the extract in a dose-dependent manner from 10µg/mL to 1000µg/mL as shown in Figure-2. The cystone drug at a dosage of 1000µg/mL was used as a positive control.

For CaOx crystal nucleation, the percentage inhibition shown by the *Terminalia arjuna* bark AE at 10µg/mL, 25µg/mL and 50µg/mL were found to be 20.8±3.6%, 27.3±2.8% and 31.1±2.5% respectively. As the concentration of AE was increased to 100µg/mL, the percentage inhibition increased to 42.63±1.4%. The inhibition was almost constant in the range of 45–50% at 200µg/mL and 500µg/mL and increased upto 61.35±0.6% at 1000µg/mL when compared to the control (with no plant extract). Addition of 1000µg/mL cystone resulted in a nucleation percentage inhibition of 57.53±3.5%.

For CaOx crystal aggregation, the percentage inhibition shown by the *Terminalia arjuna* bark AE at 10µg/mL was found to be 31.05±5.3 % which remained almost constant at 25µg/mL and 50µg/mL. As the concentration of AE was increased to 100µg/mL, 200µg/mL and 500µg/mL, the percentage inhibition increased to 34.47±2.7%, 39.9±2.3% and 44.6±2.4% respectively. The percentage inhibition increased to 49.8±2.4% at 1000µg/mL when compared to the control (with no plant extract). Addition of 1000µg/mL cystone resulted in an aggregation percentage inhibition of 69.7±4.2%.

#### CaOx crystal growth assay

**Figure 3:**
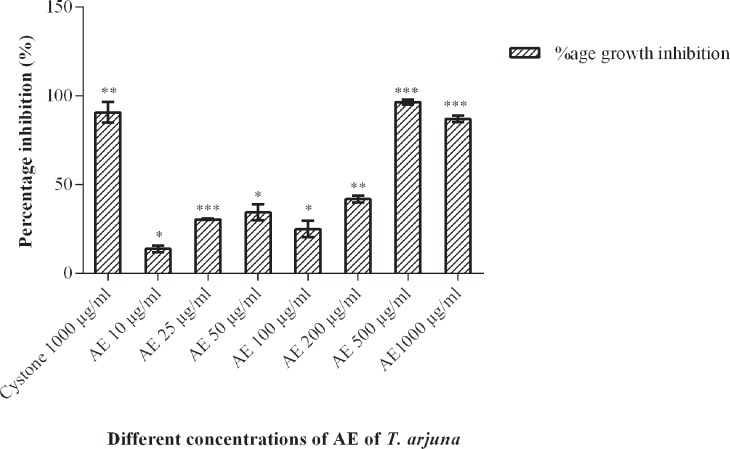
Effect of aqueous extract of Terminalia arjuna on growth of calcium oxalate crystals. Data are mean±S.D of three independent observations. *p<0.05, **p<0.005, ***p<0.0005

The AE of *Terminalia arjuna* bark showed the inhibitory effect on the growth of CaOx crystals as shown in Figure-3. When compared to the control (with no plant extract), the percentage inhibition at 10µg/mL was found to be 13.8±1.9%, which increased to 30.4±0.4% and 34.34±4.5% at 25µg/mL and 50µg/mL AE respectively. As the concentration of AE was increased to 100µg/mL, the percentage inhibition decreased to 24.97±4.6%. The percentage inhibition was significantly increased to 41.82±2.03% and 96.4±1.4% at 200µg/mL and 500µg/mL respectively and again reduced to 86.89±1.9% at 1000µg/mL. The cystone drug at a dosage of 1000µg/mL was used as a positive control. Addition of 1000µg/mL cystone resulted in a growth percentage inhibition of 90.67±5.9%.

#### Image Analysis of CaOx Crystal Morphology

**Figure 4:**
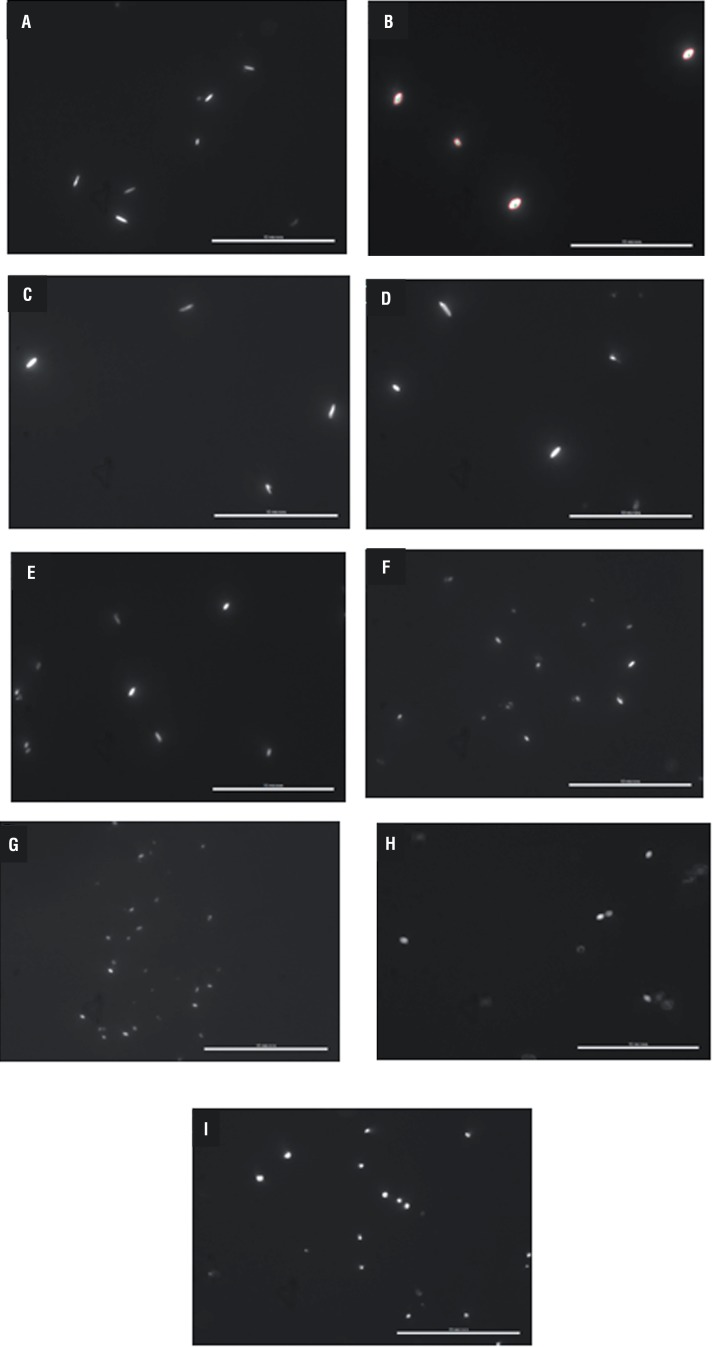
The calcium oxalate crystals, observed under upright microscope (100x), formed in the metastable solution of calcium oxalate in the absence (A) and the presence of (B) cystone (1000µg/mL) and Terminalia arjuna bark aqueous extract, (C-I) 10, 25, 50, 100, 200, 500 and 1000µg/mL.

The incubation of metastable solutions of 4.25mM calcium and 0.75mM oxalate resulted in the formation of CaOx crystals composed predominately of hexagonal CaOx monohydrate as shown in Figure-4A. Microphotography studies showed that the AE of *Terminalia arjuna* bark resulted in the formation of rounded CaOx crystals. When compared to control (with no plant sample), the AE of *Terminalia arjuna* bark modified the morphology of CaOx crystals from hexagonal to spherical shape with increasing concentrations of AE as shown in Figure 4A-I and reduced the dimensions such as area, perimeter, length and width of CaOx crystals in a dose dependent manner as shown in Table-1. The CaOx crystal area, perimeter, length and width was reduced from 0.3µm^2^, 2.31µm, 0.85µm and 0.54µm to 0.06µm^2^, 0.85µm, 0.31µm and 0.23µm by the addition of 1000µg/mL of AE respectively. The cystone drug at a dosage of 1000µg/mL was used as a positive control. Addition of 1000µg/mL cystone decreased the area, perimeter, length and width of CaOx crystals to 0.23µm^2^, 1.73µm, 0.64µm and 0.41µm respectively.

**Table 1:** Effect of Terminalia arjuna aqueous extract on the morphology and dimension of calcium oxalate crystals in vitro at concentrations of 10, 25, 50, 100, 200, 500 and 1000µg/mL as compared to control having Millipore water instead of aqueous extract. Data are mean±S.D of three independent observations.

	Control	Cystone 1000µg/mL	AE 10µg/mL	AE 25µg/mL	AE 50µg/mL	AE 100µg/mL	AE 200µg/mL	AE 500µg/mL	AE 1000µg/mL
Area (µm^2^)	0.30±0.05	0.23±0.14	0.25±0.03	0.24±0.06	0.12±0.02	0.09±0.03	0.06±0.07	0.06±0.002	0.06±0.001
Perimeter (µm)	2.31±0.21	1.73±0.72	2.04±0.26	1.92±0.24	1.25±0.2	1.02±0.19	0.84±0.07	0.92±0.11	0.85±0.007
Length (µm)	0.85±0.0	0.64±0.29	0.88±0.13	0.84±0.11	0.51±0.07	0.41±0.07	0.32±0.007	0.35±0.026	0.31±0.01
Width (µm)	0.54±0.07	0.41±0.15	0.35±0.02	0.32±0.05	0.26±0.05	0.23±0.04	0.20±0.006	0.22±0.017	0.23±0.02
Shape	Hexagonal	Hexagonal with rounded edges	Hexagonal	Hexagonal with rounded edges	Hexagonal with rounded edges	Hexagonal with rounded edges	Hexagonal with rounded edges	Hexagonal with rounded edges	Spherical

## DISCUSSION

Hyperoxaluria is a major risk factor for CaOx nephrolithiasis, which in turn is associated with renal injury. High level of oxalate causes a variety of changes in the renal epithelial cells, such as an increase in free radical production and a decrease in antioxidant status, followed by cell injury and cell death. These changes are significant predisposing factors for the facilitation of crystal adherence and retention (19). Oxalate induced toxicity and free radical production are attenuated in vivo (20) and in vitro (21) by antioxidants.

A dramatic advancement in using phytotherapy for urolithiasis treatments has been observed in the recent years and many investigators have proposed scientific studies on its efficacy. Many medicinal plants have been used since ages to treat urinary stones though the rationale behind their use is not well established. One such unexplored plant is *Terminalia arjuna*, commonly called as ‘arjuna’ or ‘arjun’. The plant is said to be a divine medicine in Vedas and has a special mention in Charaka Samhita, though not much scientific study has been done to explore the antiurolithiatic potency of *Terminalia arjuna*, which has been established through ethnobotanical studies (10). In view of its medicinal use, *Terminalia arjuna* bark extract was studied to evaluate its antiurolithiatic potential using different in vitro models.

*Terminalia arjuna* bark AE possess very high antioxidant activity due to the presence of Terpenoids. It has the ability to scavenge the free radicals with an IC_50_ of 13.11µg/mL. Thus, pretreatment with antioxidants can block oxalate induced increases in ceramide (22). Antioxidant treatments also block oxalate-induced cell death (21), suggesting a role for oxidant stress in these responses.

The effect of *Terminalia arjuna* bark extract on CaOx crystallization kinetics was studied by the time course measurement of turbidity. In this study, AE inhibited the CaC_2_O_4_ crystal aggregation in a concentration-dependent manner, similar to cystone drug, a well-known drug formulated by the Himalaya to cure kidney stones and widely clinically used for the management of urolithiasis.

In the microscopic study, AE modified CaOx monohydrate crystal morphology. A similar change in the morphology of CaOx monohydrate crystals has been previously reported with citrate and Mg^2+^ (23). Microphotography studies verified that AE of *Terminalia arjuna* resulted in the formation of round CaOx crystals. COM and COD are the major forms found in most urinary calculi. AE of *Terminalia arjuna* inhibited the growth of COM crystals, prevented the aggregation of COM crystals, and induced the formation of spherical COM crystals. These spherical COM crystals are thermodynamically less stable phase and have weaker affinity for cell membranes than hexagonal COM crystals. Both *Terminalia arjuna* AE (1mg/mL) and cystone (1mg/mL) resulted in the shape changes of CaOx crystals, as shown in Figures 4B and 4I; a more rounded polygonal crystals shape. This shape may prevent the formation of kidney stones, because crystals with this shape are more easily excreted in the urine compared with the COM.

Formation of crystals along urinary tract, driven by urinary supersaturation, is a primary requisition for the subsequent stone formation (24), although crystal formation does not necessarily lead to stone formation. Researchers have identified crystal retention as a critical step for the formation of clinically symptomatic stone from a free particle. Various physiological inhibitors of urolithiasis found in urine including citrate have been shown to decrease the saturation of CaC_2_O_4_ and inhibit crystal nucleation, growth and aggregation, while reduced crystallization inhibiting capacity of urine can play a role in stone formation (3). Interference with crystal growth and aggregation therefore seems a possible therapeutic strategy for the prevention of recurrent stone disease.

*Terminalia arjuna* bark extract is previously reported to inhibit CaOx crystal precipitation and growth (25). These results were verified in previous studies and also showed the change in the shape CaOx crystals in the presence of *Terminalia arjuna* bark AE. Recently, several plants including Herniaria hirsuta (26), Tribulus terrestris (5), Terminalia chebula (27) and Bergenia ligulata (17), are being explored for their antiurolithiatic property on the basis of their usage in the traditional medicine. An extract of H. hirsuta increased the CaOx crystal number but decreased their size. It also promoted the formation of CaOx dihydrate crystals, despite the presence of CaOx monohydrate particles (26). B. ligulata is a widely used plant in South Asia, mainly India and Pakistan, as a traditional medicine for the treatment of urolithiasis. The crude aqueous-methanolic extract of B. ligulata rhizome (BLR) was studied using in vitro and in vivo methods and the extract showed the antiurolithic activity through CaOx crystal inhibition, diuretic, hypermagneseuric and antioxidant effects (17). Very recently, in our lab, antilithiatic potency of Dolichos biflorus (28) and Trachyspermum ammi (29) has been evaluated in vitro and in vivo. Antilithiatic proteins were identified and characterized from these plants adding a new vista to study therapeutic proteins from plants.

## CONCLUSION

This study demonstrated that AE of *Terminalia arjuna* possess a high antioxidant activity and an ability to inhibit the CaOx crystallization in vitro. In addition, this extract changed the morphology and reduced the dimensions of hexagonal COM crystals to spherical COM crystals. This shape may prevent the formation of kidney stones. In the light of these studies, *Terminalia arjuna* can be regarded as a promising candidate from natural plant sources of antilithiatic and antioxidant activity with high value.

## ABBREVIATIONS

CaOx: Calcium oxalate
AE: Aqueous extract
T. arjuna: *Terminalia arjuna*
COM: Calcium oxalate monohydrate
COD: Calcium oxalate dihydrate
ESWL: Extra corporeal shock wave lithotripsy
PCNL: Percutaneous nephrolithotomy
URS: Ureteroscopy
DPPH: 2, 2-diphenyl-1-picrylhydrazyl
